# 4100 Chronic Pain in Refugee Torture Survivors

**DOI:** 10.1017/cts.2020.114

**Published:** 2020-07-29

**Authors:** Gunisha Kaur

**Affiliations:** 1 Weill Cornell Medicine

## Abstract

OBJECTIVES/GOALS: An estimated 87% of torture survivors, or 27 million people globally, suffer from chronic pain such as brachial plexopathy from upper extremity suspension or lumbosacral plexus injury from leg hyperextension. However, a vast majority of pain is undetected by evaluators due to a lack of diagnostic tools and confounding psychiatric illness. This diagnostic gap results in exclusive psychological treatment rather than multimodal therapies, substantially limiting rehabilitation, placing vulnerable individuals at higher risk of drug abuse, and increasing healthcare expenditures. We hypothesized that the novel application in torture survivors of a validated pain screen can supplement the UNIP and improve its sensitivity for pain from approximately 15% to 90%, as compared to the reference standard. METHODS/STUDY POPULATION: In this prospective, blind comparison to gold standard study, 25 patients who survived torture by World Medical Association definition first received the standard evaluation protocol for torture survivors (United Nations Istanbul Protocol, UNIP) by a trained evaluator, and subsequently received a validated pain screen (Brief Pain Inventory Short Form, BPISF) followed by a non-invasive examination by a pain specialist physician (reference standard). The primary outcome was the diagnostic and treatment capability of the standard protocol (index test) versus the validated pain screen (BPISF), as compared to the reference standard. RESULTS/ANTICIPATED RESULTS: Providers using only the UNIP detected and treated pain in a maximum of 16% of patients as compared to 85% of patients who were diagnosed with pain by the reference standard. When employed, the validated pain screen had a sensitivity of 100% [95% CI: 72% - 100%] and a negative predictive value of 100%, as compared to a sensitivity of 24% [95% CI: 8% - 50%] and negative predictive value of 19% by the index test. The difference in the sensitivity of the UNIP as compared to the BPISF was significant, with p < 0.001. DISCUSSION/SIGNIFICANCE OF IMPACT : These data indicate that the current global standard assessment of torture survivors, the United Nations Istanbul Protocol, should be supplemented by the use of a validated pain screen to increase the accuracy of chronic pain diagnosis. This would change the standard medical assessment of 70.8 million people globally, a number that continues to rise by nearly 45,000 people each day. ClinicalTrials.gov protocol number NCT03018782.

